# HPG-DHunter: an ultrafast, friendly tool for DMR detection and visualization

**DOI:** 10.1186/s12859-020-03634-y

**Published:** 2020-07-06

**Authors:** Lisardo Fernández, Mariano Pérez, Ricardo Olanda, Juan M. Orduña, Joan Marquez-Molins

**Affiliations:** 1 Departamento de Informática, Universidad de Valencia, Avda. Universidad, s/n, Burjassot (Valencia), Spain; 2 I2SysBio, CSIC-Universidad de Valencia, Cat. Agustín Escardino, Paterna (Valencia), Spain

**Keywords:** High performance computing, DNA methylation, Wavelet transform, GPU computing

## Abstract

**Background:**

Software tools for analyzing DNA methylation do not provide graphical results which can be easily identified, but huge text files containing the alignment of the samples and their methylation status at a resolution of base pairs. There have been proposed different tools and methods for finding Differentially Methylated Regions (DMRs) among different samples, but the execution time required by these tools is large, and the visualization of their results is far from being interactive. Additionally, these methods show more accurate results when identifying simulated DM regions that are long and have small within-group variation, but they have low concordance when used with real datasets, probably due to the different approaches they use for DMR identification. Thus, a tool which automatically detects DMRs among different samples and interactively visualizes DMRs at different scales (from a bunch to ten of millions of DNA locations) can be the key for shortening the DNA methylation analysis process in many studies.

**Results:**

In this paper, we propose a software tool based on the wavelet transform. This mathematical tool allows the fast automatic DMR detection by simple comparison of different signals at different resolution levels. Also, it allows an interactive visualization of the DMRs found at different resolution levels. The tool is publicly available at https://grev-uv.github.io/, and it is part of a complete suite of tools which allow to carry out the complete process of DNA alignment and methylation analysis, creation of methylation maps of the whole genome, and the detection and visualization of DMRs between different samples.

**Conclusions:**

The validation of the developed software tool shows similar concordance with other well-known and extended tools when used with real and synthetic data. The batch mode of the tool is capable of automatically detecting the existing DMRs for half (twelve) of the human chromosomes between two sets of six samples (whose.csv files after the alignment and mapping procedures have an aggregated size of 108 Gigabytes) in around three hours and a half. When compared to other well-known tools, HPG-DHunter only requires around 15% of the execution time required by other tools for detecting the DMRs.

## Background

Bisulphite-treated DNA methylation analysis requires specific treatment of DNA that modifies its sequence, as well as software tools for its analysis. Bisulphite treatment converts unmethylated cytosines (Cs) into thymines (Ts), which gives rise to C-to-T changes in DNA sequence after sequencing, while leaving methylated cytosines (5mCs) unchanged. By aligning and comparing bisulphite sequencing reads to the genomic DNA sequence, it is possible to infer DNA methylation patterns at base pair-resolution ([1]). Hydroxymethylated samples can also be obtained from different methods, the Ten-eleven translocation (TET) Assisted Bisulfite Sequencing (TAB-Seq) ([2, 3]), which produces Ts in methylated and unmethylated Cs and maintains as C the hydroxymethylated Cs (5hmC), and the oxidative bisulphate sequencing (oxBs-seq) ([4]). Different software tools have been proposed for DNA methylation analysis like RRBSMAP ([5]), the widely extended tool Bismark ([6]), or the most recent tools HPG-Methyl ([7, 8]). These tools provide single-base information of the alignment and the methylation status of each input sequence (or read). However, all these software tools yield the results as text files which usually have sizes of tens of Gigabytes, and follow the Sequence Alignment/Map (SAM) format or the Binary Alignment Map (BAM) format. Other tools like HPG-HMapper ([9, 10]) uses the methylation information of each base for each read present in the BAM files to build a DNA methylation map which gives information about the methylation level for each base of the reference genome. This map is yielded as one csv file for each chromosome in the species. Biomedical researches then have to compare the methylation level information in these files at different scales (DNA segments, CpG islands or coding regions, DNA chromosomes, etc.) besides the base-pair resolution, comparing also the results coming from different samples. Thus, many different tools for processing, displaying the methylation results and discovering differentially methylated regions (DMRs) have been proposed ([11–16]). However, most of these tools are based on statistical techniques, adding a high computational workload when applied to huge BAM or SAM files. As a result, the execution time required by these tools is large, adding an excessive delay from the moment of the sample extraction from the DNA sequencer until the moment when significant information is provided to the doctor or biomedical researcher. Also, the visualization of these data is far from being interactive. Another disadvantage of these tools is that many of them are essentially R scripts, which requires programming skills from the user. Finally, as some comparative study shows [17], these methods show more accurate results when identifying simulated DM regions that are long and have small within-group variation, but they have low concordance, probably due to the different approaches they have used for DM identification. Thus, they yield very low concordance when used with real data.

In a previous work, we proposed the building of a methylation signal and the implementation of its wavelet transform in the GPU for visualization purposes ([18]). In this paper, we present HPG-DHunter, a user-friendly graphical tool not only for interactive visualization of methylation signals but also for automatic DMR detection, based on the information generated by the Discrete Wavelet Transform (DWT). This software tool can detect and display at different scales DMRs from different samples, and it is publicly available at https://grev-uv.github.io/. The tool is based on transforming the methylation (and/or hydroxymethylation) level information of each base in the reference genome into a methylation signal, and applying the Discrete Wavelet Transform to this signal in order to detect DMRs in the Transform space. DWT performs local averaging of the methylation signal at different resolution levels, like smoothing-based approaches do ([16]). On the one hand, this averaging takes into account the biological variability present in the information gathered in the methylation maps. On the other hand, this local averaging yields a more accurate measurement of the methylation level for wider DNA segments. However, by using a dyadic decomposition of the DWT this strategy achieves a logarithmic reduction in the number of values used for comparing signals and detecting DMRs, compared to the ones used in other smoothing-based methods like [11, 14]), in such a way that the computational workload required for detecting DMRs is reduced in orders of magnitude. The validation of the tool is a comparison study, carried out with a real dataset with known DMRs, which compares the results yielded with this tool to the ones yielded by other three widely used tools, namely BSmooth [11], DSS-Single [14], and methylKit [19]. This validation study with a real dataset shows that the proposed tool is as valid and effective as the other well-known tools.

HPG-DHunter can efficiently detect and display DMRs for different thresholds of methylation rate, coverage and number of samples, since the use of GPUs allows the parallel processing of methylation signals and their interactive visualization at different scales. To the best of our knowledge, this tool is the first one that can automatically detect DMRs between samples in all the chromosomes for a given difference threshold. Moreover, it is the first tool that allows an interactive visualization of DMRs at different levels of detail. The performance evaluation results show that the batch mode of the tool is capable of detecting the DMRs in 12 chromosomes between two sets of six (human) samples (whose methylation maps have an aggregated size of 108 Gigabytes) in around three hours and a half. When compared with the other three software tools, HPG-DHunter only requires around 15% of the execution times required by any of them. These results show that the tool offers biomedical researchers a valuable tool for easily finding hyper or hypomethylation differences in any set of fastq files.

**Fig. 1:**
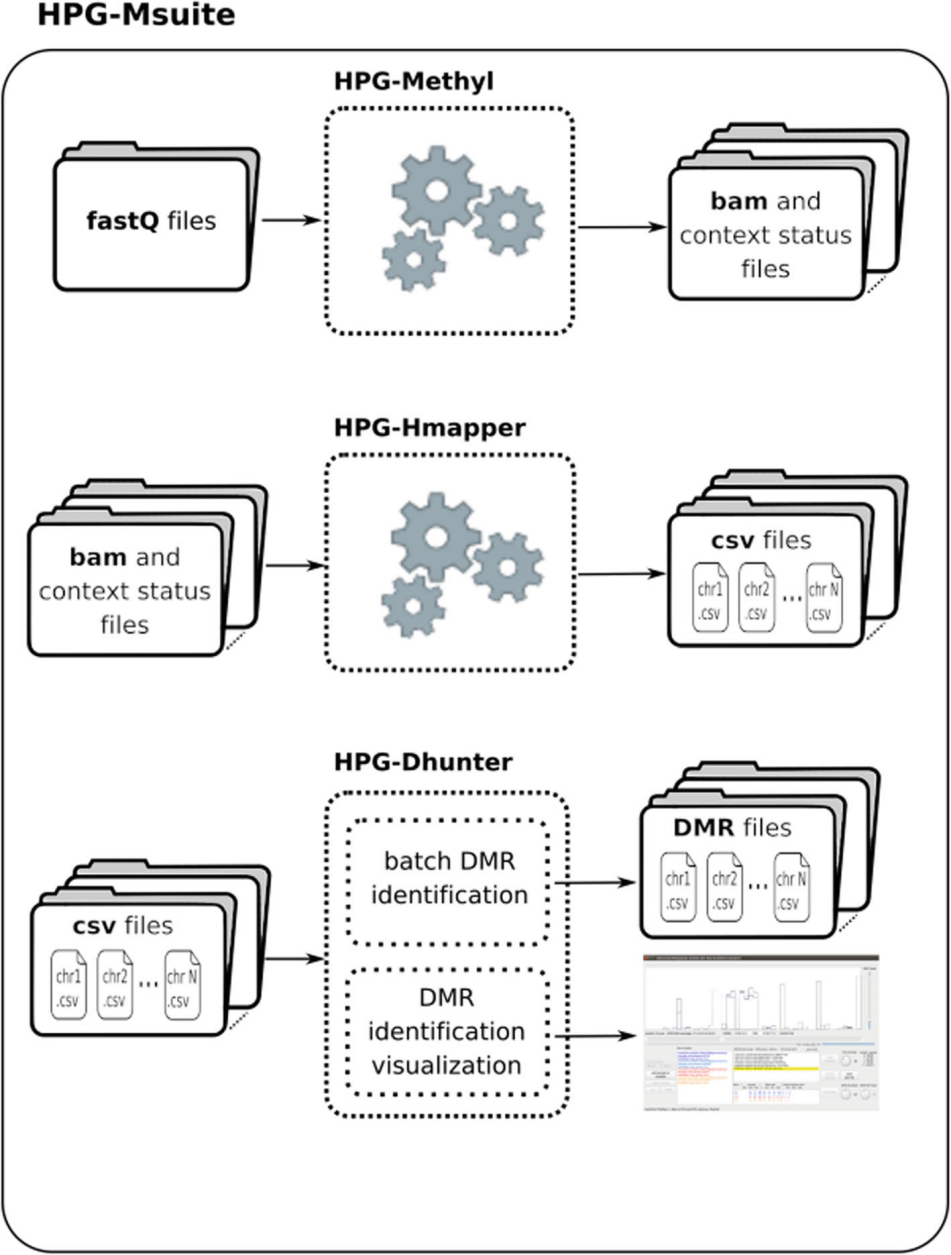
Workflow for the use of HPG-Msuite, from the fastq file to the DMR detection

HPG-DHunter is the third and last module of HPG-Msuite (https://grev-uv.github.io/), whose two first modules are HPG-Methyl ([7, 8]) and HPG-HMapper([9, 10]). This comprehensive suite carries out the whole process of methylation (and/or hydroxymethylation) analysis of fastq files coming from different samples, mapping the reads on the reference genome and building a methylation map, and detecting and visualizing DMRs between the different samples. In this way, we provide biomedical researchers with a complete solution for studying DNA hyper and hypomethylation of any species. Figure 1 shows the pipeline workflow of the HPG-Msuite. The first module is HPG-Methyl ([7, 8]), which takes fastq files as input and produces bam or sam files with the methylation and alignment data. The second module is HPG-HMapper ([9, 10]), which takes the bam/sam files as input data and produces one csv file for each chromosome and strand. Finally, HPG-DHunter takes the csv files (one for each chromosome and strand) and builds a methylation signal showing the methylation level for each location in the chromosome. Moreover, it can load and compare the methylation maps coming from different samples and build different methylation signals, detecting and displaying the DMRs among them. The rest of the paper describes in detail the implementation of HPG-DHunter and its performance evaluation with real datasets.

## Implementation

**Fig. 2:**
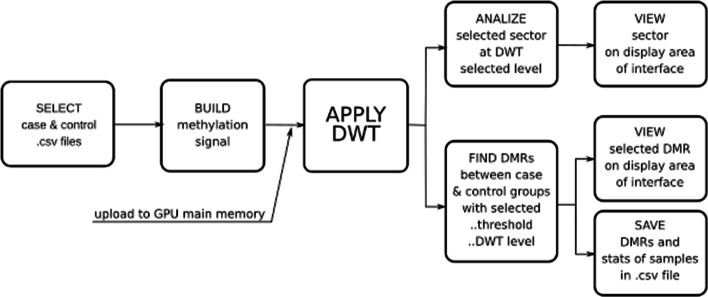
Workflow for the use of HPG-DHunter

HPG-DHunter takes as input files the csv files (one for each chromosome and individual/sample to be analyzed), and it produces as output a csv file for each chromosome, containing the DMRs found between the selected individuals/samples. The workflow of our approach is illustrated in Fig. 2. This Figure shows that HPG-DHunter first read the files corresponding to the case and control samples (the user selects each file). Then, each methylation signal is built and the DMR detection is carried out by processing the methylation signal with the Haar Wavelet Transform (the DWT is applied to each signal). At this point, the tool provides a visualization interface which allows the visual analysis at different scales of any of the DMRs found, and the results of the DMR detection can be saved in -csv files.

The building of the methylation signal and the application of the wavelet transform to that signal are described in a previous work [18]. However, in order to make this paper self-contained, we also summarize here these steps in the two next subsections.

### Building the methylation signal

The first step consists of reading the.csv files containing the mapped samples, and computing the methylation ratio of each DNA base. It must be noted that any alignment and/or mapping tool can be used to produce these.csv files, although we have used HPG-Methyl and HPG-Mapper for the alignment and mapping procedures, respectively.

**Fig. 3:**
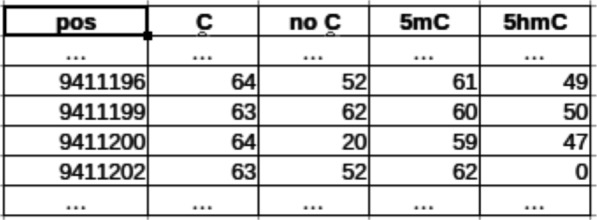
Information present in the csv files

Figure 3 shows the information that should be available in the input csv files for each DNA location where methylation is detected (there should be a csv file for each chromosome and each sample). For each location in the chromosome where a methylated cytosine has been found, it counts the number of reads which have been found having a cytosine, methylated cytosine, hydroxymethylated cytosine or a base different from a cytosine aligned to that particular location. The values in column labeled as “C” shows the number of reads containing a non-methylated cytosine in that location. The values in the column labeled as “5mC” show the number of reads which have been found having a methylated cytosine. These values (the values in the column labeled as “5mC”) include the number of reads which have been found having a hydroxymethylated cytosine (column labeled as “5hmC”). It must be noted that the samples with a base different from a cytosine (values in the column labeled as “no C” in Fig. 3) should be ignored in the DMR search procedure ([11, 14]). We will have one file to be read per sample to be studied (usually there are several control patients and several case patients, but there can be also studies which analyzes different tissues from the same patient, or other cases). In the rest of the paper, we will assume that the study to be carried out focuses on a given chromosome (a given csv file) and different control and case patients.

Different specific issues must be taken into account when trying to identify DMRs ([15]). First, there exists a strong spatial correlation among methylated or hydroxymethylated CpG islands, thus in these regions there is no need for the same coverage levels as in other regions. Second, the coverage, sequencing depth, or number of reads aligned on each position, is very important to properly weight the variability of the analyzed samples. Finally, the heterogeneity in the methylation levels of different tissues, or even among cells of the same tissue, may lead to errors in the identification of DMRs.

Taking into account these considerations, in our work we have defined the methylation level for each cytosine in the reference genome as the methylation ratio or percentage of methylated reads *mratio* for each position *k*, computed as:

1 $$ mratio_{k} \; = \; \frac{5mC_{k}}{C_{k}+5mC_{k}}  $$

where 5*mC*_*k*_ is the number of reads containing a methylated cytosine which have been aligned onto that DNA position *k*, and *C*_*k*_ is the number of read containing non-methylated cytosines which have been aligned onto that position (as shown in Fig. 3). In the same way, we have computed the hydroxymethylation ratio *hmratio* for each position *k* as:

2 $$ hmratio_{k} \; = \; \frac{5hmC_{k}}{C_{k}+5hmC_{k}}  $$

where 5*hmC*_*k*_ is the number of reads containing a hydroxymethylated cytosine which have been aligned onto that DNA position *k*. One of the advantages of this approach based on ratio is that it takes into account the biological variability among the different samples processed through bisulphite sequencing. Nevertheless, due to the errors inherent to the bisulphite treatment, the files can contain samples not belonging to the target regions of the chemical sensors. Thus, we could find for example some reads in the csv file with a value of 2 in the “5mC” column and also a value of 2 in the “C” column, for a coverage ratio *mratio*_*k*_=1/2. Since the same ratio would result for a target region with a value of 200 in both columns, we must establish a minimum coverage threshold for filtering the reads in the csv file. This coverage threshold should be established by the user through a selector knob available in the proposed tool.

Once the methylation ratio has been computed, the methylation signal should be built. Although the same process can also be carried out for the hydroxymethylation signal, for the sake of clarity we are going to exclusively describe the computation of the methylation signal. Since a methylation signal *X* is estimated to follow a beta-binomial distribution ([11, 14]), we have computed each element *x*[*k*] of the methylation signal *X* as

3 $$  x[\!k] \; = \; mratio_{k}  $$

It must be noted that *x*[*k*] ranges between 0 and 1.

### Wavelet transform of the methylation signal

Once the methylation signal *X* is built, the Discrete Wavelet Transform is iteratively applied on the methylation signal, until the desired resolution level is reached. We have implemented a dyadic multiresolution decomposition of the signal by iteratively applying the following filtering algorithm:

4 $$  c_{j+1}[\!k] \; = \; \sum\limits_{l} h[\!2k-l] \: c_{j}[l]  $$

5 $$  d_{j+1}[\!k] \; = \; \sum\limits_{l} g[\!2k-l] \: c_{j}[l]  $$

where *c*_*j*_[ *k*] and *d*_*j*_[ *k*] are the scaling and detail coefficients at resolution *j*, respectively, *g* highpass decomposition filter of the wavelet transform, and *h* is the lowpass decomposition for the selected Discrete Wavelet Transform. It must be noted that Eqs. 4 and 5 provide the scaling coefficients *c*[*k*] and the detail coefficients *d*[ *k*] at *j*+1 level, starting in both cases from the scaling coefficients *c*[*k*] at *j* level. Both equations include a downsampling procedure. In particular, Eq. 4 reduces the size of *c*_*j*+1_[ *k*] to half of the size it had in level *j*. Also, it must be noted that for *j*=0, *c*_0_[ *k*]=*x*[ *k*].

It must be noted that any orthogonal or bi-orthogonal wavelet base can be used for the implementation of *h* and *g* filters. Thus, the validation study (see the “Tool validation” section) considers different filters, whose values are not shown here for the sake of shortness. As a representative example, we show here the filters implemented when the HPG-DHunter module uses the Haar Discrete Wavelet Transform. The reason is that this wavelet transform has a reduced compact support (*h* and *g* filters are of length equal to 2), which on the one hand results in short computation times and on the other hand allows an efficient and simple GPU implementation. Concretely, the *g* and *h* filters for the Haar Wavelet Transform are the following ones:

6 $$  g[l] \; = \; \left[-\sqrt{2}/2, \: \sqrt{2}/2\right]  $$

7 $$  h[l] \; = \; \left[ \sqrt{2}/2, \: \sqrt{2}/2\right]  $$

**Fig. 4:**
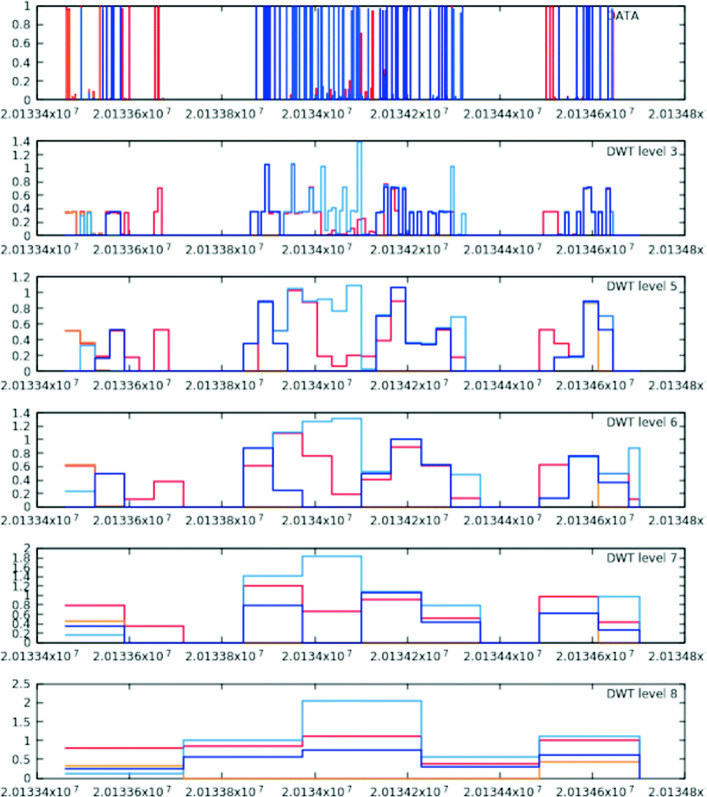
Segments of 1241 DNA positions, coming from control and case methylation signals, displayed with different resolution levels. Each colored line corresponds to a methylation signal

Starting from the original control and case signals, the different versions of the methylation signals (with different resolution levels) are built by iteratively applying the DWT to the current signals. As an illustrating example, Fig. 4 shows two methylation signals processed with the proposed approach, such as they are displayed by HPG-DHunter. The two colored lines correspond to the two methylation signals. The plots in the top part of this Figure show the original methylation signals built from a DNA segment of 1241 nucleotides (from position 47830931 to position 47832172), while the rest of the plots downwards (starting from the plot below the original signals) show the same methylation signals iteratively built in the second, fourth, sixth, eighth and tenth iterations of the Haar transform. All these plots show on the X-axis the location of each base in the chromosome which is being displayed (in this case we are using human samples). On the Y-axis, they show the *c*_*j*_[ *k*] values.

Figure 4 illustrates the main advantage of this approach: each iteration reduces by one-half the number of different values in the signal, since they are computed as the average value of two signal values in the previous iteration. As a result, the signal has a lower resolution but the comparison of the signal with the signals coming from other samples adds a much lower computational workload, and it is easier to detect significantly different values. Thus, the signals in the bottom plot have only 5 different values (the 1241 DNA locations take are only 5 different methylation values).

**Fig. 5:**
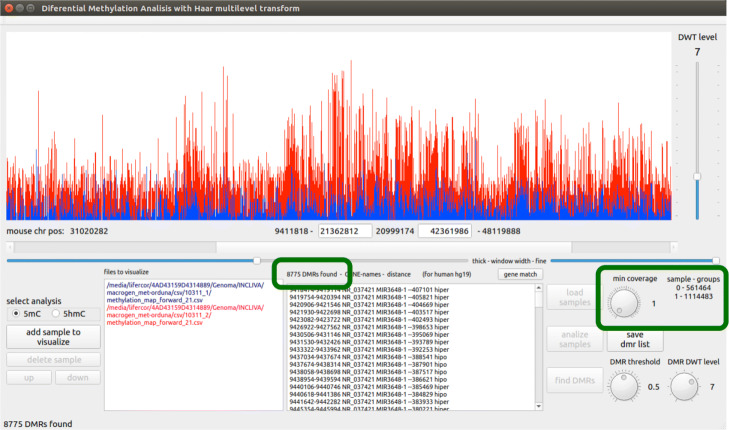
Example methylation signals for a coverage threshold of 1 as visualized in the tool interface. A lot of DMRs (8775) are found because the minimum coverage is used

**Fig. 6:**
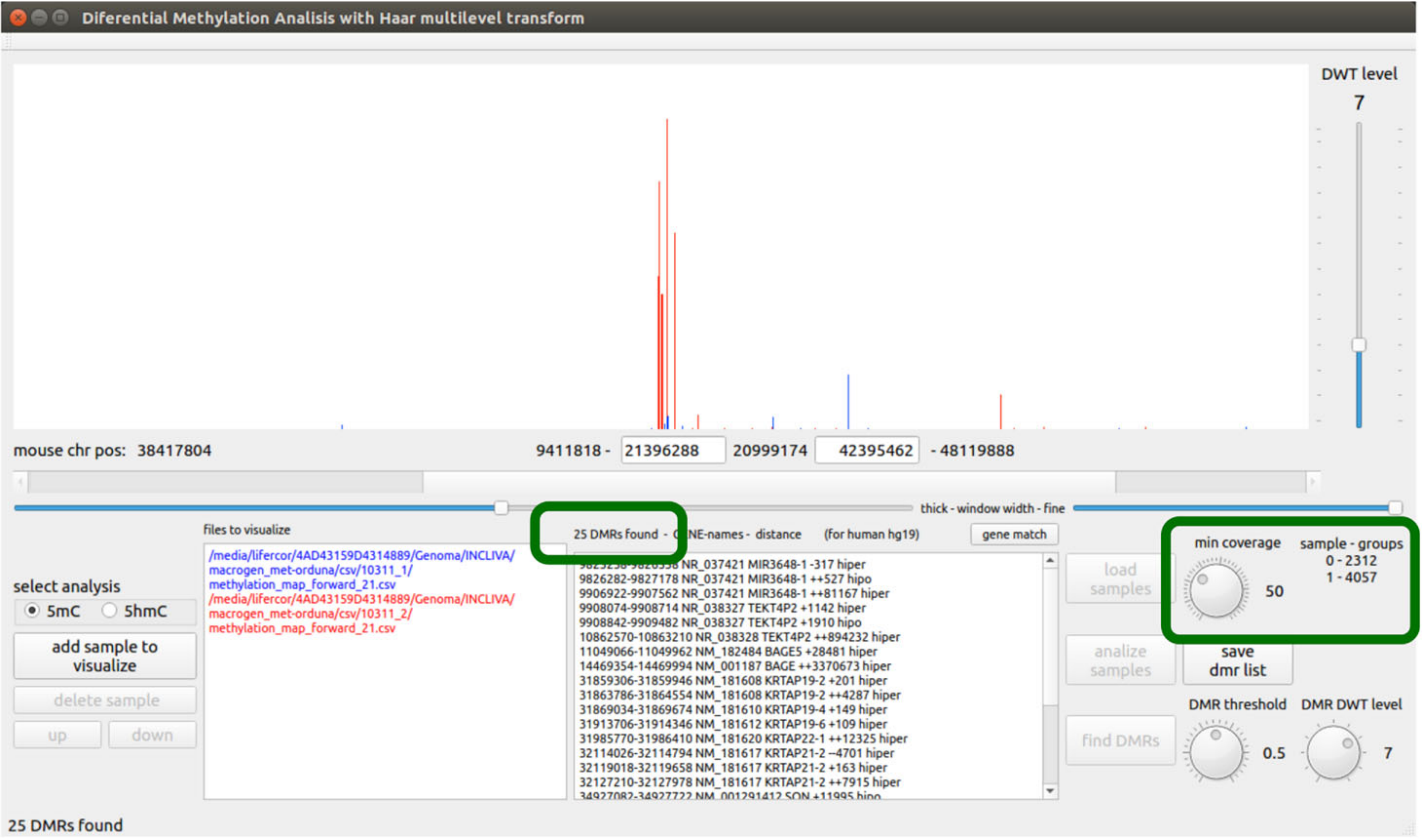
Example methylation signals for a coverage threshold of 50 as visualized in the tool interface. A few (25) DMRs are found because all the locations not arriving to the minimum coverage are not considered

In order to build the methylation signal, enough memory space should be allocated for containing an array with as many elements as adjacent locations exists between the initial and final DNA location to be analyzed (multiplied by the number of samples to be analyzed). The elements of the array corresponding to locations with non-zero values are filled with the corresponding $v^{0}_{j}$ values, and the whole array is copied to the GPU memory, where the next steps are carried out. Figure 5 shows an example of two methylation signals where the coverage threshold has been set to 1, while Fig. 6 shows the resulting methylation signals coming from the same csv files when the coverage threshold is set to 50.

HPG-DHunter stores the methylation signals in the GPU memory to carry out the two main tasks: detection of DMRs and the visualization of the methylation signals. Each step of the wavelet transform decomposes the signal into two halves, the first one containing a low resolution version of the signal with half the number of points, an the second one containing the wavelet coefficients. Thus, after each iterative step of the wavelet transform, for DMR detection purposes we only keep in the GPU memory the first half of the signal. This half will become as the input signal for the next iterative step of the DWT. Therefore, once the original signal is transferred to the GPU memory, no additional GPU memory is required for performing the whole DWT iterative process.

### Interactive DMR detection

The DMR detection is carried out by simply comparing the values of different transformed signals with the same level of transformation. A methylation threshold should be selected by the user (using the graphical interface described below) to determine if two or more different signals should be considered as differentially methylated. All the methylation signals are iteratively transformed until the selected transformation level is reached. Then, the DMR detection is carried out in three stages: the first one consists of computing the average methylation value of all the considered signals in each group (case and control samples) for each DNA position. The second step is to find the difference (in absolute values) between these methylation average values. If this difference exceeds the threshold selected by the user through the interface, then that position is marked as DM. Finally, the third step consists in grouping adjacent DM locations.

**Fig. 7:**
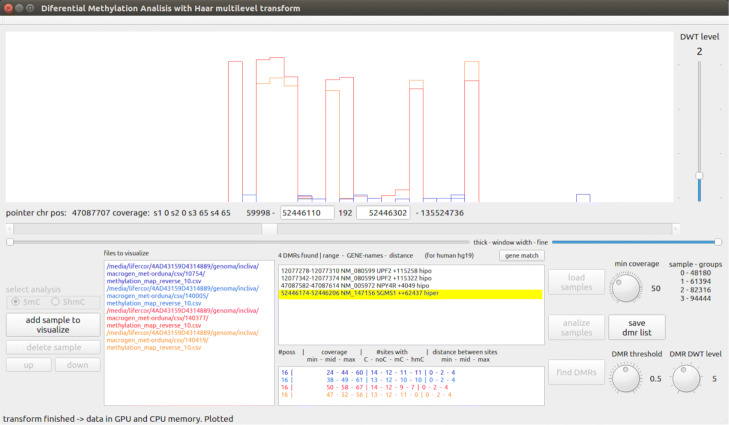
Example of a DMR identification and visualization in the tool interface. The name of each input file is given a color corresponding to its signal representation, and the DMR range, including the gene name, is shown in the central window

Thus, the validation process for DMR detection consists of the following process: for each value *x*_*j*_[*k*] (each value *k* of the signal in the *j*th transformation level), we check that (by default) at least 25% of the 2^*j*^ values in the original methylation signal (the values *x*_*j*_[*k*]⋯*x*_*j*_[*k*+2^*j*^−1] which have contributed to the value *k* of the signal in the *j*th transformation level) have a coverage ratio higher than the coverage threshold set by the user. In turn, this default percentage of 25% can be tuned by the user. In positive case, that position *x*_*j*_[ *k*] is kept marked as DM. Otherwise, it is not marked as DM. It must be noted that if this validation process was carried out in the building stage of the methylation signal (by filtering those *x*_0_[ *k*] values lower than the coverage threshold) then many values of the methylation signal would have been changed to zero, significantly changing the signal at all the transformation levels. By applying the validation process after the DMR detection process, we guarantee that the methylation signal is faithfully computed with respect to the methylation values in the csv input files, and so it is the DMR detection process. Figure 7 shows an example of DMR identification and visualization with a coverage threshold of 50 reads.

It must be noted that in each transformation level each signal point represents the weighted methylation value of several DNA locations. The selection of the most appropriate transformation level to be used for detecting DMRs is left to the user criterion, who can easily repeat all the computations and visualization of the results by moving up and down the DWT level selector (see the “DMR visualization” section). The selected level of wavelet transformation has an effect in the width of the DMRs detected, in such a way that 2 elevated at the selected transformation level will determine the minimum DMR length which can be detected in this level. Therefore, the user should select the level of DMR transformation based on the minimum DMR length which is being searched. The transformation level used in the results section has been the 5th level in the real dataset (and the 4th level in the case of the synthetic dataset) because we experimentally tried different levels for considered input dataset, and we found that this was the best level. Nevertheless, it must be noted that the same happens with the main parameters of the other considered tools, there is no a priori default value which yields the best results for all the input datasets. HPG-DHunter uses the csv files delivered by HPG.Mapper as input data. Thus, the sample size cannot affect the tool sensitivity, since the tool always builds a methylation signal for the whole genome. If the samples are greater, the methylation signal is built in a more robust manner (with more information about each DNA location). The sample size can only affect to the time required by aligners like Bismarck or HPG-Methyl.

The tool interface allows, by clicking on any item of the list of DMRs found, to open the Ensemble web page (http://www.ensembl.org) in a navigator window and display the corresponding genes and other information in the reference genome for that location, in order to help biomedical researchers to identify the most significant DMRs for their study. Although the tool is configured for the human reference genome, it can be easily tailored to show the reference genome for other species.

**Fig. 8:**
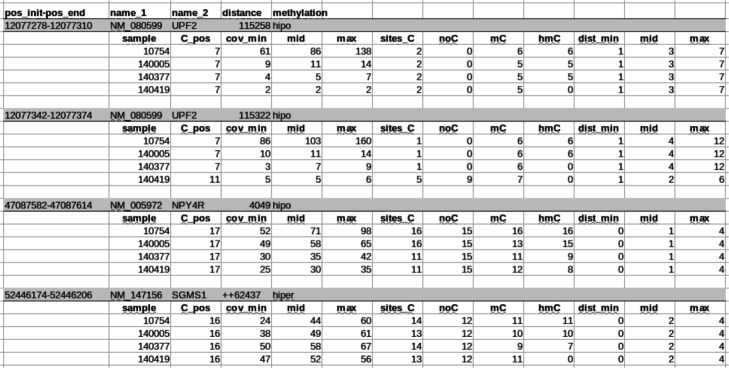
Example csv output file

Once the DMRs have been identified in the selected samples, the results can be stored in a different CSV file for each chromosome. For each DMR found, this file shows two levels of details. The first one shows the initial and final position of the DMR, the most common names of the closest gene to that position, the distance between the DMR and this gene, and whether the methylation difference is in favor of the cases or the control samples (hyper/hypo). The second level of detail shows basic statistics for each of the analyzed samples. Each line starts with the name of the sample, the name of nucleotides affected in that DMR, the minimum, average and maximum coverage of reads, the number of reads detecting Cs, mC, hmC and other nucleotide different from C, as well as the minimum, average and maximum distance between locations with information. Figure 8 shows an example of an output csv file (also a.gif format file can be selected).

Therefore, the analysis procedure for detecting DMRs with HPG-DHunter is composed of the following steps:

1. Selection of the samples (csv files) to be analyzed.
2. Selection of the minimum coverage threshold.
3. Selection of the transformation level. At this point, the tool can detect and display a list of DMRs.
4. Depending on the results, any of the previous parameters can be tuned, and the whole process could be repeated.

### DMR visualization

After the DMR detection has been carried out, the results should be displayed in a comprehensive and clear way. HPG-DHunter allows the interactive visualization of methylation signals corresponding to any DNA segment at different levels of detail. In particular, those segments where DMRs have been previously detected by the tool. The visualization process uses the data generated by the DWT, and it is completely carried out in the GPU, as described in our previous work ([18]).

We have designed a graphical interface for HPG-DHunter which allows to carry out the whole process of DMR detection and visualization in an intuitive and friendly manner: it allows to select the sample files to be analyzed, the type of analysis to be performed (5mC or 5hmC), and the parameters of this analysis: the minimum coverage ratio the samples should have to be taken into account, the DWT level to be applied to the signals, and the DMR threshold. Figure 7 shows a snapshot of this graphical tool. The top part of the tool is devoted to graphically displaying one of the DMRs found, while the bottom part of the tool contains the controls to select the input files and analysis parameters, as well as a window for listing the DMRs found in text mode. On the bottom-left corner, it shows the buttons for selecting the type of analysis and the sample input files to be analyzed (each file path appears in a different color, the same one used in the top window for displaying its corresponding signal). Next to the input files box is the DMR results box, which lists the DMR(s) found and if the case group is hyper or hypo methylated. At the right of the results box there are the buttons for loading, analyzing (carrying out the DMR detection) and saving the DMR list found for a given analysis (it must be noted that different analysis with different DMR thresholds and DWT levels may be performed on the same sample files), as well as knobs and sliders for zoom-in and out, and interactively exploring the adjacent regions to the DMRs. Figure 7 shows an example of identification and visualization carried out by HPG-DHunter for a case of one control sample and one case sample, for a coverage threshold of 50 reads, a difference methylation threshold of 0.5, and a transformation level (DWT level selector) of 2. On top of the results window, we can see that with these parameters 8775 DMRs have been found.

### Batch DMR detection

HPG-DHunter also allows the automatic DMR detection without user intervention, analyzing a selection of chromosomes (or all of them). That is, the tool can yield an output file like the one in Fig. 8 for all the chromosomes for both a case and a control group, studying all the samples.

**Fig. 9:**
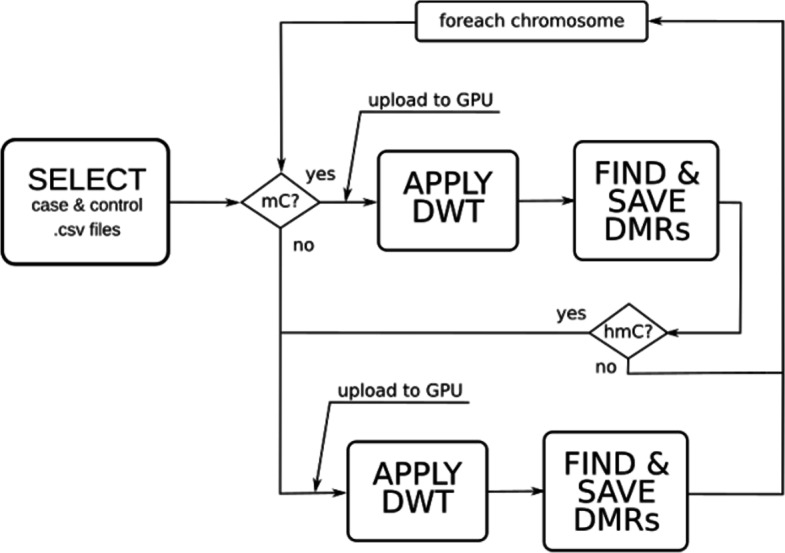
Pipeline procedure for batch DMR detection

Figure 9 shows the pipeline corresponding to the batch DMR detection procedure. Once the analysis parameters and input files are selected by the user, this procedure iterates over each one of the selected chromosomes. However, since the slowest task is the file read and the building of the methylation signal, the procedures creates as many threads as number of chromosomes (csv files) to be read. Then, the wavelet transform is computed (at the selected transform level) in the GPU. This process is carried out in a batch manner, processing file chunks which are adequate for the GPU memory size. As the GPU computing finishes, the resulting data of each iteration is stored in CPU memory, freeing GPU memory for a new batch file. The DMR detection takes place in the CPU as explained in the “Interactive DMR detection” section. That is, grouping the samples in control and cases and filtering those differences between both groups which are greater than the selected threshold. The results are stored in output files and both GPU and CPU memory are freed up for processing the next chromosome.

Thus, in the case of batch DMR detection the following procedure should be carried out:

1. Selection of the samples (csv files) to be analyzed, grouped in *case* and *control*.
2. Selection of the type of methylation to be analyzed (mC or hmC).
3. Selection of the DNA thread to be used in the analysis (forward and/or reverse).
4. Selection of the minimum DMR threshold to be detected.
5. Selection of the transformation level on which DMRs will be detected.
6. Selection of the chromosomes to be analyzed. Analysis in batch mode.
7. Inspection of the DMRs found using the visualization interface.
8. Depending on the results, any of the previous parameters can be tuned, and the whole process could be repeated.

**Fig. 10:**
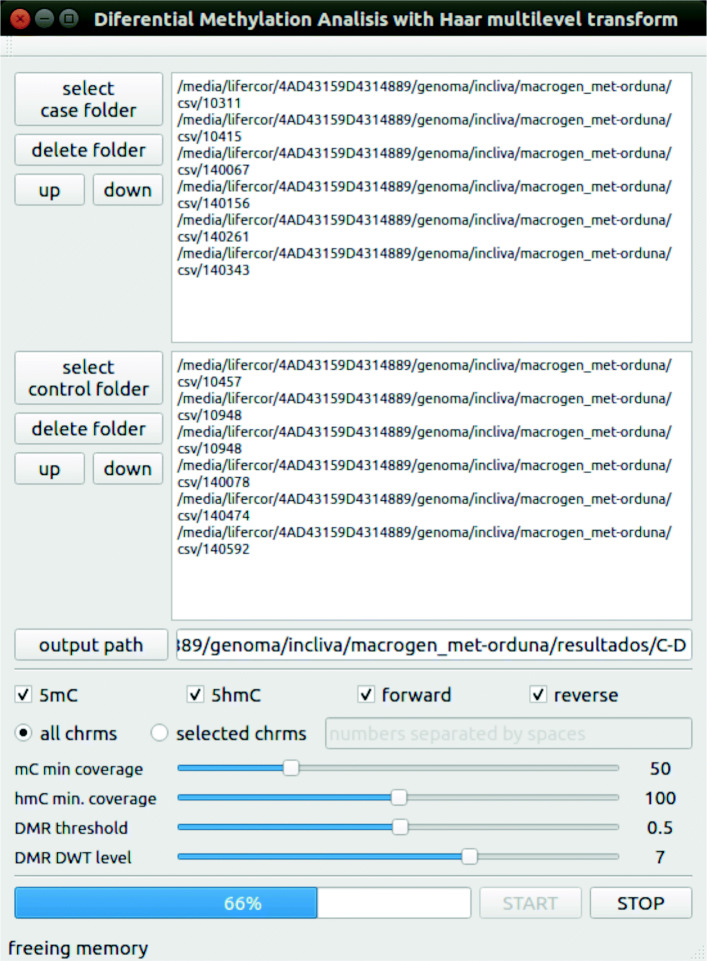
Example snapshot of the tool designed for batch DMR detection

In order to help the user in all these tasks, we have developed a visual interface with all these parameters implemented as knobs, sliders, buttons or selectors. Figure 10 shows a snapshot of this tool, at the exact moment when the DMR batch tool is processing 66% of the selected input files, for a dataset comprising six control input directories and six case input directories. In each directory there are as many files as the number of chromosomes considered for the study (a button allows to select to study all the chromosomes or only a subset (selecting each chromosome number)). The output path selects the output directory where the tool will store a file for each chromosome and study (5mC or 5hmC) containing the DMRs found (the same data which can be seen in the visualization tool and the associated output files, including the hyper/hypomethylation diagnosis and the closest gene).

However, the GPU memory size also restricts the number of output files to be displayed. Depending on the chromosome length, HPG-DHunter allows the displaying of a range from 2 to 10 (human) chromosomes. When used for DMR search in other species, the maximum number of chromosomes displayed may vary. Thus, a feasible way of studying DMRs is the previous batch analysis of the considered chromosomes and then the visualization of those areas of special interest from the output csv files. In this way, the tool can analyze the DMRs found in all the chromosomes, although it can simultaneously display the DMRs for only some of them.

## Results

### Tool validation

Some comparative studies [17] show that the tools based on statistical techniques and validated with statistical significance studies like BSmooth [11] or DSS-Single [14] show more accurate results when identifying simulated DM regions that are long and have small within-group variation, but they have low concordance, probably due to the different approaches they have used for DM identification. As a result, they provide very different results when used with real datasets, and usually there is no a clear set of “correct” DMRs in these datasets. Effectively, we first tested BSmooth [11], DSS-Single [14], and methylKit [19] with the same real datasets, but they yielded many different DMRs among them, without a clear set of DMRs to be detected in order to validate our tool.

Thus, in order to validate the proposed tool, we have not performed a statistical significance study, as other tools like BSmooth [11] or DSS-Single [14] do. Instead, we have used the NBCI’s Gene Expression Omnibus with reference GEO GSE 124610 dataset, coming from a study about the effects of ascorbic acid on the hydroxymethylation of carcinoma renal cells [20]. The interest of this real dataset for our validation study is based on the known hydroxymethylation levels in the different samples, as well as the existence of some genes associated to suppressive effects of the cell tumoral action (that is, some specific DMRs should be detected in these genes). Thus, we can check if the detection and visualization of the hydroxymethylation levels at different scales correspond to the results shown in that work [20]. We have focused on several genes in different chromosomes identified as very important by the referenced study [20]. Also, we have tested the tools on a synthetic dataset.

#### Selection of the wavelet transform

For validation purposes, we have considered in our tool not only the Haar wavelet transform used in [18], but other wavelet transforms: the Bi orthonormal 3.1, 3.3, and 3.5 transforms [21], as well as the Spline wavelet transform [22]. The idea is to test the tool with several different transforms, and select the one which detects all the known DMRs while detecting the less number of false positives.

We have established fixed values for a set of parameters (regardless they are adequate or not from a biological point of view), and we have used them for all the considered wavelet transforms. The fixed values are: 6th level of wavelet transformation (windows containing 64 DNA positions), minimum coverage of 100 samples, DMR identification threshold of 0.25, and a ratio of 7% of positions with minimum coverage per window (4 positions with minimum coverage in each 64 position window).

Table 1 shows if each of the considered transform has detected (x) or not (-) DMRs within the most important genes (NBPF1, AKT3, KMT2C, and DOCK8), where the reference study [20] assumes that it should appear DMRs. Also, this table shows the total number of DMRs detected in chromosome 7. These results show that the Haar wavelet transform obtain the best results, since it detects the DMRs in the four genes. The Biorthogonal 3.1 and the Spline 2.2 transforms do not detect the DMRs in the AKT3 nor in the KMT2C genes. However, the Biorthogonal 3.1 detects almost double the number of DMRs detected by the Haar transform, suggesting that this wavelet transform detects a number of false positives. Thus, we have considered the Haar and the Spline 2.2 as the two best options.

**Table 1:** DMR detection in genes NBPF1, AKT3 (chrom. 1), KMT2C (chrom. 7), and DOCK8 (chrom. 9), as well as total number of DMRs detected

wavelet type	NBPF1	AKT3	KMT2C	DOCK8	#DMRs
HAAR	X	X	X	X	446
BIOR 3.1	X	-	-	X	805
BIOR 3.3	X	-	-	-	172
BIOR 3.5	X	-	-	-	291
SPLINE 2.2	X	-	-	X	107

Next, starting from the results shown in the first test we have setup a second detection test, using a wider set of genes described as important in the referenced study [20] and testing exclusively the Haar and the Spline 2.2 wavelet transforms. This time we have decreased the DMR identification threshold to 0.1, in order to increase the number of DMRs detected. Table 2 shows the results for this test.

**Table 2:** Second DMR identification test, using HAAR and SPLINE 2.2 wavelet transforms

chr	gene	haar	spline	#DMRs haar	#DMRs spline
1	NBPF1	X	X	2737	617
1	AKT3	X	-	2737	617
4	CWH43	X	X	2101	956
7	KMT2C	X	-	1695	886
8	ZHX1	-	-	1481	732
9	DOCK8	X	X	1028	319
10	PRKG1	X	X	1175	561
10	MXI1	-	-	1175	561
15	SMAD6	-	X	605	308

Table 2 shows that there are differences in the DMR detection for the tested genes, since the Haar transform detects DMRs in six of the nine genes, while the Spline 2.2 transform detects DMRs in five of the nine genes. Since we must select one wavelet transform to comparatively test our tool with other tools, we have selected the one which seems to provide the best results for these real data. Thus, we have used the Haar wavelet transform as the default wavelet used by HPG-DHunter for DMR detection.

#### Comparison with other tools using a real dataset

Next, we have performed a comparison study for detecting DMRs in the same real dataset [20] using other three tools: BSmooth [11], DSS-Single [14], and methylKit [19]. Each of these tools include a number of parameters which can be tuned by the researcher, depending on the dataset to be used and the target of the research carried out. In order to make a fair comparison and an effective validation, we have followed two different approaches: first, we have tuned the main parameter for each tool (the one establishing the threshold difference between two samples to consider it as a DMR) until each tool has detected the DMRs in all the nine genes cited in the renal carcinoma study [20]. Then, we have studied the DMRs found by each tool. Previously, the dataset was filtered to obtain a minimum coverage of 50 reads for all the samples. For the second comparative approach, we have tuned the main parameter value for each tool until it has led to the detection of a similar number of genes with detected DMRs in all the tools (hundreds).

Concretely, for the first approach the BSmooth parameter *Cutoff* had to be varied from 4 to 0.005. For DSS-Single, the *Threshold* parameter had to be varied from 3 to 0.01. For methylKit, the *Diff* parameter ranged from 25 to 0.5, and the *qvalue* ranged from 0.5 to 0.01. Finally, the HPG-DHunter *Threshold* parameter ranged from 0.4 to 0.01, and the DWT levels used for this study were 6 and 5 (although the results shown here were the ones with the 6th. transformation level because they were better than the ones with the 5th transformation level). Table 3 shows the values for these parameters that had to be set in order for each tool to detect the known DMRs in each chromosome. The methylKit column shows both the Diff / qvalue pair of values set in each case. In order to show how far from its default values each tool has been tuned, the last three rows show the average value computed from the previous seven values, their standard deviation, and the default value used by the tool.

**Table 3:** Parameter values set for finding the known DMRs

Chrom.	BSmooth	DSS-Single	methylKit	HPG-DHunter
chr1	1	0,5	25 / 0.01	0.40
chr4	0,75	1	5 / 0.5	0.25
chr7	4	0,5	15 / 0.01	0.35
chr8	0,1	0,5	5 / 0.5	0.07
chr9	1	0,5	15 / 0.01	0.40
chr10	0.5	1	17 / 0.01	0.40
chr15	0.1	1	3 / 0.4	0.06
Avg.	1.06	0.71	12.14% / 0.24	0.255
Std. Dev	1.35	0.27	8.07% / 0.25	0.16
Default	2	1.00E-05	25% / 0.01	0.25

Table 3 show that the DMRs in chromosomes 8 and 15 are the most difficult ones to be detected, since all the tools require for these chromosomes the most extreme changes from the default values in order to detect them. Also, the last three rows show that HPG-DHunter and BSmooth have default values which fall within the interval [*avg*±*std*.*dev*], indicating that it has not been necessary to significantly change the default values in these tools to make them detect the known DMRs. However, both methylkit and DSS-Single have some parameters whose default value is far from the required value to make these tools detect the known DMRs. This issue makes more difficult for the user to properly tune these tools for detecting DMRs which are difficult to detect.

Table 4 shows the results yielded by each of the considered tools in raw numbers, as well as some indicative percentages. The first row shows the total number of DMRs detected by each tool, denoted as A. The second row, denoted as B, shows the number of these DMRs which have been detected within the limits of a gene. The third row, denoted as C, shows the number of genes in which a DMR has been detected by each tool. The rest of the rows show, in terms of percentage, the ratios B/A and C/A, in order to measure the behavior of the tools.

**Table 4:** Number of DMRs and different percentages of genes when the tools are tuned to detect DMRs in the known genes

	BSmooth	DSS-Single	methylKit	HPG-DHunter
A-Total DMRs	10717	9022	51074	8595
B-DMRs in genes	5515	4576	18177	3863
C-genes with DMRs	1594	2245	2422	1886
%DMRs in Genes(B/A)	51.46	50.72	35.59	44.94
%Genes in DMRs tota(C/A)	14.87	24.88	4.74	21.94
%Genes in (DMRs in gen)(C/B)	28.90	49.06	13.32	48.82

The first row of Table 4 shows that HPG-DHunter detects the lowest number of DMRs, but the numbers in the third row show that the number of genes in which DMRs have been found is not very different among the considered tools, suggesting that those DMRs found in other places can be false positives. It is worth mention that the number of genes in which HPG-DHunter has found DMRs is within the range of the other three tools. The percentages shown in the last rows show that again the percentages yielded by the proposed tool are also within the range yielded by the other tools. Finally, it should be highlighted that HPG-DHunter and DSS-Single obtain the best percentages (last two rows), with the important difference that, as shown in Table 3, it is much more difficult to tune DSS-Single than HPG-DHunter to obtain these results.

Next, we have studied the concordance of the considered tools. Table 5 shows the amount of identical genes where DMRs have been detected by some subsets of the considered tools, in order to measure if these tools detected the same DMRS or not. We have denoted each tool by the first letter of his name. Thus, *B* correspond to BSmooth, *D* denotes DSS-Single, *M* stands for methylKit, and *H* denotes HPG-DHunter. When any of these letter shows an overline, it means the complementary set. That is, the column labeled as B-D-M-$\overline {H}$ shows the number of DMRs which have been detected by BSmooth, DSS-Single and methylKit and have not been detected by HPG-DHunter.

**Table 5:** Number of matching genes with DMRs for different subsets of tools

Chrom.	B-D-M-H	B-D-M-$\overline {H}$	B-D-$\overline {M}$-H	B-$\overline {D}$-M-H	$\overline {B}$-D-M-H
chr1	133	42	15	21	54
chr4	157	38	1	1	125
chr7	23	10	0	5	100
chr8	137	5	1	137	4
chr9	51	27	0	14	39
chr10	89	69	7	2	31
chr15	265	19	5	9	30
TOTAL	855	210	29	189	383

The mostleft column in Table 5 shows that all the four considered tools have identified 855 matching genes with DMRs. Comparing these numbers with the ones in the third row of Table 4, it can be seen that the number of matching genes with DMRs found by all the tools is around 50% of the DMRs found by BSmooth (the one with the lowest number of genes with DMRs). That is, there is a very low concordance among the DMRs found by the different tools, as predicted in other studies [17]. Also, assuming that the 855 matching genes in which all the four tools have detected DMRs are likely to be the correct DMRs, then the numbers in the third row of Table 4 indicate the number genes in which wrong DMRs or false positives have been detected (the higher number of genes with DMRs, the more false positives detected by each tool, since all of them exceed 855). In that row, the proposed tool detects the second lowest number of genes, validating the tool as a more accurate tool than methylKit or DSS-Single.

The rest of the columns in Table 5 show the number of genes in which each tool has not detected DMRs and the other three tools have done it. When comparing these columns with the third row of Table 4 we can see that, although BSmooth detects 1594 genes with DMRs and HPG-Hunter detects 1886 genes, HPG-Hunter fails to detect only 210 “correct” genes, while BSmooth fails to detect 383 “correct” genes. That is, the proposed tool detects more false positives but misses less “correct” DMRs. It is worth mention that the column corresponding to methylKit shows the lowest number of genes, and the reason for this behavior can be the remarkable number of DMRs found by this tool, as shown in the first row of Table 4. Since this tool detects five times more DMRs than the second one (detecting much more false positives), it detects DMRs in all the genes where the rest of tools also detects DMRs.

The average length of the DMRs identified in each chromosome by all the methods in the paper, as well as the absolute methylation difference between the two groups samples, are shown in Table 6. For each considered method, the column labeled as “Length” shows the average DMR length found in each chromosome, while the column labeled as “Diff.” shows the absolute methylation difference between the two groups of samples. The negative or positive numbers depend on which group shows the highest methylation. The DMR length distribution shows a very high variability not only among the considered chromosomes for a given tool, but also among the tools. For example, the average length of the DMRs found by DSS-Single in chromosome 8 is 361 nucleotides and 1222 nucleotides in chromosome 4. The average length of the DMRs found by BSmooth in these chromosome are 122 and 185 nucleotides, respectively, and the ones found by HPG-DHunter are 79 and 75, respectively. In this sense, HPG-DHunter shows the lowest variability among chromosomes. A case apart is Methylkit which, since it considers each nucleotide as a posible DMR and therefore all their DMRs have a length of 1 nucleotide (instead of a single DMR of three adjacent nucleotides, this tool yields 3 DMRs of length 1).

**Table 6:** Average DMR lengths and absolute methylation differences between the group samples

	BSmooth		DSS-Single		methylKit		HPG-DHunter	
Chrom.	Length	Diff.	Length	Diff.	Length	Diff.	Length	Diff.
1	136	12.90	392	-18.70	1	18387.35	73	-555.83
4	185	1.02	1222	-8.02	1	35655.10	75	-437.74
7	79	-2.79	373	-10.51	1	19936.31	73	-314.50
8	122	12.47	361	-6.15	1	-2379.04	79	-355.87
9	78	4.14	368	-5.69	1	27117.98	75	-242.54
10	111	-0.08	1064	-6.60	1	21333.46	72	-265.63
15	72	12.55	820	-7.26	1	25010.34	81	-285.62

For the second comparative approach (tuning the main parameter value for each tool until it has led to the detection of a similar number of genes with detected DMRs in all the tools), we finally used a value of 1.0 for the BSmooth *Cutoff* parameter. For DSS-Single, the *Threshold* parameter was 0.5. For methylKit, we used a *Diff* parameter of 25%, and a *qvalue* of 0.5. Finally, the HPG-DHunter *Threshold* parameter was 0.35. Table 7 shows the results yielded by each tool for this approach.

**Table 7:** Number of DMRs and gene percentages found when all the tools yield similar number of genes with DMRs

	BSmooth	DSS-Single	methylKit	HPG-DHunter
A-Total DMRs	7006	4552	13181	5374
B-DMRs in genes	3674	2368	6519	2493
C-genes with DMRs	1229	1536	1539	1605
%DMRs in Genes(B/A)	52.44	52.02	49.46	46.39
%Genes in DMRs total(C/A)	17.54	33.74	11.68	29.87
%Genes in (DMRs in gen)(C/B)	33.45	64.86	23.61	64.38

Table 7 shows that the tuning of each tool has achieved that the number of genes with DMRs are similar, ranging (in that order) from 1229 to 1605. For these numbers of genes with DMRs, HPG-DHunter detects the second lowest number of DMRs (first row), which indicates that this tool is as effective as the other ones (it detects a number of DMRs which is in the range of the rest of the tools).

Next, Table 8 shows the number of genes in the considered chromosomes where different subsets of tools detected DMRs, similarly to Table 5. The first row in Table 8 shows that the number of DMRs that are detected by all the tools is very low, compared to the number of DMRs detected separately (first row of Table 7). Also, this table shows that the number of genes with DMRs “missed” by HPG-DHunter is within the range of the values yielded by the other tools.

**Table 8:** Number of matching genes with DMRs for different subsets of tools

Tools	chr1	chr4	chr7	chr8	chr9	chr10	chr15	Total
B-D-M-H	140	68	78	50	47	51	41	475
B-D-M-$\overline {H}$	35	11	21	20	16	10	16	129
B-D-$\overline {M}$-H	19	16	17	10	11	6	10	89
B-$\overline {D}$-M-H	25	17	20	14	15	20	10	121
$\overline {B}$-D-M-H	67	33	32	27	29	22	35	245

Finally, Table 9 shows the number of DMRs found in the genes which are know to have DMRs [20]. The last row shows the number of these genes in which each tool has found DMRs (the number of non-zero values in that column).

**Table 9:** Number of DMRs found in the genes which are known to have DMRs

gene	BSmooth	DSS-Single	methylKit	HPG-DHunter
NBPF1	4	1	1	1
AKT3	1	2	4	2
CWH43	0	0	0	0
KMT2C	3	1	0	1
ZHX1	0	1	0	0
DOCK8	1	1	0	1
MXI1	0	0	0	3
PRKG1	0	0	5	2
SMAD6	0	0	0	0
# of matches	4	5	3	6

Table 9 indicates how effective is each tool for finding the known DMRs when they detect a similar number of DMRs, and it shows that the proposed tool is capable of detecting DMRs in the highest number of known genes. These results prove HPG-DHunter as a tool as valid and effective as the other three ones for DMR detection on real datasets.

#### Comparison with other tools using a synthetic dataset

Next, we have performed a comparison study for detecting DMRs in a synthetic dataset using the same tools. In order to generate the synthetic dataset, we have used WGBSSuite [23], an stochastic generator developed to test and compare statistical methods, as the reference tools used in this evaluation, to generate differentially methylated base pairs. The first step for building a synthetic dataset is to analyze a real dataset to extract statistical information to parameterize WGBSSuite. We have generated a dataset with two groups and two samples each one, coming from chromosome 1 of the renal carcinoma study [20]. Using these samples, WGBSSuite yielded the main statistical values shown in Table 10. Using these data for configuring the generation of the synthetic dataset, WGBSSuite generated a new dataset containing two groups (samples) of one sample (replicate) each. We introduced as input data a differential methylation threshold value of 0.3 and ten thousand methylated locations, and for these input values the WGBSSuite analysis determined a DNA segment of around 18,300,000 base pairs with 4,394 differentially methylated locations.

**Table 10:** Parameterization values yielded by WGBSSuite

Avg. diff. in methylation proportion	0.3823
Std. dev. in diff. in methylation proportion	0.1242
Max. diff. in methylation proportion	0.8092
Prob. of success in a methylated region	0.917
Prob. of success in a unmethylated region	0.095
Avg. coverage in a methylated region	53
Avg. coverage in an unmethylated region	63
Phase diff (difference between groups to validate DMR)	0.3

In order to make a fair comparison in this case, we have tuned the main parameter for each tool (the one establishing the threshold difference between two samples to consider it as a DMR) using the similar values shown in Table 3, the ones for which the reference tools obtained the best results for the real dataset.. In the case of HPG-DHunter, since the length of the DMRs this synthetic dataset can be of any length (including a single base pair), we tuned the tool for the DWT levels 2, 3, and 4, and finally was the 3rd transformation level the one which provided the best results for this tool. Also, the value of the threshold has been shifted to the range of 0.04-0.2. Changing the range of the values for the main parameter in the reference tools yielded, in general, worse results for these tools.

Table 11 shows the number of true positive DMRs found by the considered tools when each of the main parameter values is used (we consider that a true positive DMR found by a tool is a DMR covering one or more of the positions marked as a differentially methylated base pair by WGBSSuite). For each tool, Table 11 shows two columns for each considered tool. The left one shows the main parameter value used in that tool, and the right column shows the number of positive DMRs for that parameter value.

**Table 11:** True positives yielded by each tool for different parameter values and the synthetic dataset

BSmooth		DSSSingle		methylKit		HPG-DHunter	
Cutoff	DMRs	Thres	DMRs	Diff	DMRs	Thres	DMRs
4	0	1e-5	4	25	3212	0..2	146
3	0	0.01	564	20	3608	0.15	427
2	0	0.05	1544	15	3620	0.1	2369
1	61	0.1	1950	10	3620	0.06	3681
0.75	1465	0.5	3212	5	3620	0.05	3794
0.5	3396	0.75	3651	3	3620	0.04	3856
0.25	4128	1	4098				
0.1	4383						

Table 11 shows for all the tools a similar behavior to the one observed for a real dataset. All the tools except Methylkit need a significant variation of the main parameter from their default value in order to detect the greatest amount of true DMRs. One reason for this behavior can be the fact that WGBSSuite generates a single-base pattern and Methylkit is the only tool which internally works on a single-base basis.

Nevertheless, in order to make a fair comparison we also need to know the ratio of true and false positives found by each tool to yield the results in Table 11, as well as the ratio of the total differentially methylated base pairs in the synthetic dataset found by each tool. Table 12 shows these values for the considered tools for each number of true DMRs found by each tool (each value appearing in Table 11). The hit rate is shown in the columns labeled as “Hit”, and the percentage of differentially methylated base pairs in the synthetic dataset found by each tool is shown in the columns labeled as “%”. We have computed the hit ratio as the ratio of the number of true DMRs found by each tool divided by the number of total DMRs found by that tool. Thus, the ratio of false positives would be one minus the hit rate.

**Table 12:** Hit rate and percentage of DMRs in the synthetic dataset found by each tool for different parameter values

BSmooth			DSSSingle			methylKit			HPG-DHunter		
DMRs	Hit	%	DMRs	Hit	%	DMRs	Hit	%	DMRs	Hit	%
0	0	0	4	1.0	0.1	3212	1.000	73.1	146	0.942	3.3
0	0	0	564	0.815	12.8	3608	0.999	82.1	427	0.936	9.7
0	0	0	1544	0.753	35.1	3620	0.996	82.4	2369	0.945	53.9
61	0.61	1.4	1950	0.728	44.4	3620	0.996	82.4	3681	0.934	83.8
1465	0.552	33.3	3212	0.599	73.1	3620	0.996	82.4	3794	0.924	86.3
3396	0.501	77.3	3651	0.523	83.1	3620	0.996	82.4	3856	0.885	87.8
4128	0.478	93.9	4098	0.441	93.3						
4383	0.448	99.7									

Table 12 shows that BSmooth manages to detect almost all the differentially methylated base pairs in the synthetic dataset (99.7%) at the cost of detecting more the double of true DMRs (the 4383 true DMRs found are only 44.8% of the total DMRs found by the tool). Similar figures are yielded by DSS-Single, which manages to detect 4098 true DMRs (93.3% of the existing ones), but this number is only 44.1% of the total number of DMRs detected by this tool. That is, it detects more false than true positives. Methylkit shows a very different behavior. It shows a very high hit ratio for all the values of the Diff parameter, detecting a very low ratio of false positives. It also detect a high proportion of the existing DMRs in the dataset (82.4%). Finally, HPG-DHunter shows a high hit ratio for all the threshold values considered, although the percentage of DMRs in the dataset detected is low for high threshold values. Nevertheless, for low threshold values HPG-DHunter manages to find more true DMRs than Methylkit, with a slightly lower hit ratio. These results validate HPG-DHunter as a tool for DMR detection which has similar concordance with other well-known and extended tools when used with real and synthetic data.

### Performance evaluation

This section presents the performance evaluation (in terms of execution time) of the proposed tool when processing a real dataset consisting of samples coming from human patients. The data were provided by the INCLIVA Instituto Investigación Sanitaria del Hospital Clínico de Valencia (https://www.incliva.es/), for carrying out a medical study about the effects of DNA methylation in patients with Diabetes Mellitus2 (DM2). The DNA samples were extracted from four different patient groups, denoted as A, B, C and D. Group A denotes those patients who do not suffer from DM2 (control patients) and do not show resistance to insulin treatment. Group C denotes control patients (not suffering from DM2) who are resistant to insulin. Group B denotes those patients suffering from DM2 (case patients) who do not show resistance to insulin. Finally, group D denotes those patients suffering from DM2 and showing resistance to insulin. The purpose of the study is to find the DMRs among each possible pair of these groups. The dataset is composed of 96 fastq files, corresponding to six samples (six different individuals) per group. The data for each individual are stored in two fastq files (forward and reverse) for a (paired-end) methylation dataset. The aggregated size of all the fastq files coming from all the patients was 1 Terabyte.

The hardware platform used in the performance evaluation consists of a computer based on the Intel(R) Xeon(R) CPU E5-2650 v4 @ 2.20GHz processor, with 12 cores and 2 threads per core, and a 64 Gb RAM memory, as well as a GeForce GTX 1080 graphic card, with 2560 cores and 8 Gb GDDR5X RAM memory. The procedure steps implemented in the GPU have been carried out using the CUDA 10.1.105 library.

The first pre-processing task consists of the alignment and methylation analysis of the fastq files, and it has been carried out using the HPG-Methyl tool ([7, 8]), producing as many.bam files as fastq files are in the dataset. The second pre-processing task has consisted of the building of a methylation map using HPG-HMapper ([9, 10]), yielding 48.csv files, two for each chromosome (since the samples were paired-end, we have one file for the forward and other one for the reverse strand) for each sample (patient). The first step for processing the samples has been the building of the methylation signal for each sample, as described in the Implementation Section. Following the instructions of the INCLIVA medical staff, we have computed the average signal of each group as the average value of the six signals in each group, in order to take into account the variability existing among patients and/or tissues. At this point, we have used the batch DMR detection tool for computing the DWT of the signals and finding DMRs between each two average signals coming from different groups.

Table 13 shows the execution time required for computing the DWT of the signals (including the average signal) and the batch DMR detection between each possible pair of groups for each of the chromosomes shown in the mostleft column. The selection of these chromosomes was indicated by the INCLIVA staff as the most likely chromosomes to show DMRs. The total aggregated size of the csv files corresponding to these 12 chromosomes was 108.414 Mbytes, that is, around 108 Gigabytes of data. The last row shows the aggregated time (measured in minutes) required to find the DMRs in all these chromosomes. In this case, the input parameters were a DMR threshold of 0.35 and a DWT level of 6. Table 13 shows that, as it could be expected, no significant differences arise when we compare the different columns of the table, but there are some differences among the rows (chromosomes), due to their length. That is, the execution time is proportional to the chromosome length, but it is not affected by the shape and/or differences between the methylation signals. Also, this table shows that the total execution time required for computing the DWT and finding DMRs is around thirty five minutes for each pair of groups, for a total aggregated time of around three and a half hours for processing the 108 Gigabytes dataset.

**Table 13:** Execution times (ms.) required for batch DMR detection among all the groups

Chrom.	A-D	A-B	A-C	B-C	B-D	C-D
22	94399	103456	96638	112233	107983	104961
20	103958	111796	104936	120412	115308	115132
17	190331	206827	192663	221405	210298	205949
13	74378	78857	75617	79799	80081	77474
12	180999	196676	183465	203209	196508	191967
11	214391	231394	215500	245430	236118	231187
9	172293	185016	173362	194221	190247	184382
8	148693	157845	151269	162844	159958	154460
7	190588	204318	192951	214982	207414	202518
4	154287	162503	155872	165420	159543	158972
3	218035	233437	219794	246532	235429	231717
2	270559	289130	274293	302823	292595	284348
Total (min.)	33.55	36.02	33.94	37.82	36.52	35.72

For comparison purposes, we have also executed methylKit [19], BSmooth ([11]) and DSS-Single ([14]) on the same dataset. That is, we have executed these other tools using the.csv files yielded by HPG-HMapper as the input files. However, at this point, it is worth mention that methylKit performs a pre-processing step on the csv input data files, in order to adapt the data to the format required for this tool. In this pre-processing, methylKit filters those DNA locations where any of the samples do not reach the minimum coverage. As a result, the dataset actually used for DMR detection has a significantly smaller size than the input data files. On the contrary, HPG-DHunter performs the filtering based on coverage after reading the input data, when the methylation signal is built. Therefore, in order to make a fair comparison we have measured the required time for HPG-DHunter and methylKit since the instant when the csv files are started to be read until the instant when DMRs are yielded. In order to let BSmooth and DSS-Single take advantage from the pre-processing carried out by methylkit, we have used this pre-processed dataset as the input for both BSmooth and DSS-Single. Although we have compared the results for the same sample groups shown in Table 13, we show here only the results corresponding to the A-D DMR search, for the sake of shortness. The results for the rest of the groups were very similar. This dataset (the csv files for groups A and D) has a size of 16.784 Mbytes, around 16 Gigabytes.

Table 14 shows that HPG-DHunter only requires a small fraction of the execution time required by the other tools to detect the DMRs in all the considered chromosomes. Looking at the last row, we can see that the total time required by HPG-DHunter for all the considered chromosomes is around 15% of the time required by BSmooth, which is the tool with the second shortest execution time. These results validate the proposed tool as an efficient tool for batch DMR detection.

**Table 14:** Execution times (milliseconds) required for DMR detection in different tools

Chrom.	methylKit	BSmooth	DSS-Ssingle	HPG-DHunter
22	490603	492606	490508	94399
20	643062	642859	670401	103958
17	990127	989304	991321	190331
13	726562	725685	724213	74378
12	1229539	1220503	1221206	180999
11	1279281	1273718	1274000	214391
9	1164930	1161675	1173081	172293
8	958367	957953	953994	148693
7	1401475	1394688	1449790	190588
4	1420670	1402048	1449776	154287
3	1581448	1574495	1575470	218035
2	2034938	2017989	2196166	270559
Total (min.)	232.02	230.89	236.17	33.55

## Conclusions and future work

In this paper, we have proposed a new tool named HPG-DHunter, a software tool for detecting and visualizing DMR from different samples. The tool is based on transforming the methylation information existing in the CSV files into a methylation signal, and using the Wavelet transform to process this signal in such a way that the computational workload required for detecting DMRs is reduced in orders of magnitude in regard to other methods. HPG-DHunter can efficiently detect and display DMRs for different methylation thresholds, coverage and number of samples, since the use of the GPU allows the parallel processing of methylation signals and their interactive visualization at different scales.

The validation study using real and synthetic data has shown, in a comparison study with other well-known and extended tools, the good balance between accuracy and sensibility of HPG-DHunter. The performance evaluation results shows that the batch mode of the tool is capable of automatically detecting the existing DMRs for half (twelve) of the human chromosomes between two sets of six samples (whose.csv files after the alignment and mapping procedures have an aggregated size of 108 Gigabytes) in around three hours and a half. When compared to other well-known tools, HPG-DHunter only requires around 15% of the execution time required by other tools for detecting the DMRs.

These results show that the proposed tool offers biomedical researchers a valuable tool for easily finding hyper or hypomethylation differences in any set of fastq files.

As a future work to be done, we plan to implement the proposed tool as an Internet-based service, in such a way the user uploads the fastq files, our server computes the DMRs in batch mode, and later the user can interactively visualize the results through a web interface. Also, we plan to add DMR visualization functionalities, including boxplot of methylation levels between groups in one or several DMRs, heatmap of DNA methylation levels of a list of DMRs, genes, pathways etc..

## Availability and requirements

Project name: HPG-Dhunter Project home page: https://github.com/grev-uv/hpg-dhunter. Operating system: Linux Ubuntu 16.0 LTS or higher. Programming language: C. Other requirements: 64 bit Intel CPU compatible with SSE4.2, Nvidia driver (v384 or higher), CUDA API (v9 or higher). License: GNU GPLv3. Restrictions to use by non-academics: non applicable.

## Data Availability

The source code for the proposed tool HPG-Dhunter is publicly available from the Github repository at https://grev-uv.github.io/, together with the installation instructions and the system requirements.

## Abbreviations

DMR: Differentially Methylated Region
